# The Role of Hyperbaric Oxygen Therapy in Pneumatosis Cystoides Intestinalis—A Scoping Review

**DOI:** 10.3389/fmed.2021.601872

**Published:** 2021-02-17

**Authors:** Diogo Alpuim Costa, Pedro Modas Daniel, João Vieira Branco

**Affiliations:** ^1^Centro de Medicina Subaquática e Hiperbárica (CMSH), Portuguese Navy, Lisbon, Portugal; ^2^Centro de Investigação Naval (CINAV), Base Naval Do Alfeite, Portuguese Navy, Almada, Portugal; ^3^CUF Oncology, Haematology and Oncology Department, Lisbon, Portugal; ^4^NOVA Medical School, Faculdade de Ciências Médicas, Lisbon, Portugal

**Keywords:** pneumatosis cystoides intestinalis, pneumatosis intestinalis, pneumatosis coli, hyperbaric oxygen therapy, hyperbaric oxygen, oxygen, treatment, review

## Abstract

Pneumatosis cystoides intestinalis (PCI) is characterized by gas-filled cysts within gastrointestinal tract wall from esophagus to rectum, with preferential involvement of large and small intestine. PCI is rare with an estimated incidence of 0.03 to 0–2% in general population. PCI can be distinguished into idiopathic (15%) or secondary (85%) and the clinical picture ranges from completely asymptomatic to life-threatening intraabdominal complications. Although etiology of PCI appears to be multifactorial, the exact pathophysiology is poorly understood and two main theories have been proposed (mechanical and bacterial). Over the last decades, an enormous therapeutic armamentarium was considered in PCI's management, including hyperbaric oxygen therapy (HBOT). Treatment comprises conservative treatment in mild cases to surgery in highly symptomatic and complicated PCI. In the late 70s, HBOT started to be used in selected cases of PCI not responding to conservative measures. Since then, several case reports, case series, and reviews have been published in the literature with variable outcomes. The overall response rate and complete response were 92.1% (*n* = 82/89) and 65.2% (*n* = 58/89), respectively, with a median follow-up of 7 months. Furthermore, HBOT is extremely safe, with few reported complications in the literature when used for PCI. Nevertheless, a randomized, controlled, and double-blind clinical trial is unlikely to occur given the rarity of PCI, logistical issues of HBOT, and methodological considerations related to adequate blinding with a sham-controlled group. HBOT in combination with personalized diet and antibiotics may be beneficial for moderate to severe PCI in patients with no indication for emergency exploratory laparotomy. The purpose of this article is to synthesize the existing data, analyse results of previous studies, identify gaps in knowledge, and discuss PCI' management, including the proposal of an algorithm, with a special focus on HBOT.

## Background

Pneumatosis cystoides intestinalis (PCI) was first described by Johann George Du Vernoi (also known as Du Vernoy) in an autopsy specimen in 1730 (1691–1759). The pioneering work was published in the Commentaries of the Imperial Academy of Science of Petersburg for the years 1730–1731 and printed in 1738, entitled as “*Aer intestinorum tam sub extima quam intima tunica inclusus: observationes anatomicae*” (1). In 1825, Mayer coined the name of the disease as “*pneumatosis cystoides intestinorum*,” which was adopted by several authors (2). In 1899, Hahn reported the first PCI in a living person (3). In the literature PCI is described under different denominations, including pneumatosis intestinalis, pneumatosis coli, cystic lymphopneumatosis, peritoneal lymphopneumatosis, intestinal emphysema, intestinal bullous emphysema, pseudolipomatosis, gas cysts of the intestines, and intraluminal gas (4, 5).

The overall incidence of PCI is not precisely known, with some studies showing rates of 0.03 (two cases among 6,553 patients in serial autopsy studies) to 0.2% (two cases in 1,900 examinations) (5–9). Furthermore, PCI detection seems to be increasing with therapeutic arsenal expansion and a higher number of complementary diagnostic exams and surgical procedures performed (10). Nevertheless, the exact rate is not possible to determine as many asymptomatic cases are beyond the clinical scope (5). PCI in adults typically presents in the fourth to eight decades (4, 10, 11). In an Asiatic database systematic review, the peak age at onset was 45.3 ± 15.6 (ranged, 2–81) years, male to female ratio of 2.4:1 and the average disease course of 6 months (11).

PCI is characterized by gas-filled cysts within gastrointestinal tract wall from esophagus to rectum, with preferential involvement of the large and small intestine (4, 5, 8–12). Cysts may be confined to mucosa, submucosa, or subserosa, or involve all the three layers. Subserous cysts are most frequently observed in small intestinal pneumatosis, while submucous cysts in colonic pneumatosis (12).

Although etiology of PCI appears to be multifactorial, the exact pathophysiology is poorly understood and two main theories have been proposed (mechanical and bacterial) (4, 5, 8–11). PCI can be distinguished into idiopathic or secondary and the clinical picture ranges from completely asymptomatic to life-threatening intraabdominal complications (4, 5, 8–10, 13). PCI's management is generally conservative for patients with mild to moderate symptoms and invasive procedures are reserved for those refractories to medical therapy or who have developed complications (4, 5, 9, 10, 13).

Hyperbaric oxygen therapy (HBOT) started to be used in selected cases of PCI not responding to conservative measures (5, 14). The rationale for HBOT clinical benefit in PCI is related to the dissolution of the gas-filled cysts and the antimicrobial activity, particularly for anaerobes gas-producing bacteria (5, 14, 15).

With the lack of large randomized control studies to guide the use of HBOT in the treatment of PCI, the goal of this review is to synthesize the existing data, analyse results of previous studies, identify gaps in knowledge, and discuss PCI' management, including the proposal of an algorithm, with a special focus on HBOT.

## Methods

Base on the evidence methodology of a scoping review, our aim was to map and clarify the existing published literature on the effectiveness of HBOT for the treatment of PCI. We choose the scoping review as the best methodology for our research objectives, which were to rapidly map the existing literature, chart data from the studies, and clarify concepts. We developed an a priori protocol to define our main objective and methods. No language nor date limits were applied. Search strategies included review articles, clinical practice recommendations, case series, case reports, images and supplemental files.

On January 6–8th 2021 published literature was searched through the PubMed and Google Scholar, using two appropriate controlled keywords: “pneumatosis” [MeSH] AND “hyperbaric oxygen” [MeSH]. References from selected articles were scanned in order to identify other papers.

Using Covidence (www.covidence.org), we inputted our inclusion/exclusion criteria and selected the articles independently by two reviewers (PMD, JVB).

The inclusion criteria defined included: (1) review articles of PCI; (2) articles and expert meetings reporting clinical practice recommendations for PCI' management; (3) articles reporting the physiological effects of oxygen on PCI; (4) case series of patients with PCI treated with HBOT; (5) case reports of patients with PCI treated with HBOT; (6) articles/abstracts must be available with full-text.

The exclusion criteria were: (1) ongoing study and abstracts were excluded; (2) case reports that only included normobaric oxygen; (3) case reports in which the response to HBOT had not been determined (exceptionally, data could be accepted if the PCI' therapeutic protocol had been suspended due to HBOT side effect); (4) articles not mentioning PCI and/or HBOT at all.

For each case report the information concerning patient's age and gender, underlying disease or risk factor for PCI, other (previous) treatments, HBOT protocol, overall response (improvement or resolution of symptoms), complete response (resolution of symptoms) and follow-up period were retrieved directly from the manuscript data.

Our final data managing was performed in Microsoft Excel version 16.41 (20091302) using a data charting form developed for our protocol.

## Results

Results were restricted to data available in English, French, Portuguese, Hungarian, Mandarin and Japanese. Since the majority of PCI case reports were in Japanese, the help of an interpreter was required for a full-text reading. The same was applied to an article published in Hungarian and another in Mandarin. Afterwards, the screening and selection of articles, quality assessment, and data extraction were performed independently by two reviewers (PMD, JVB), according to the pre-planned inclusion and exclusion criteria. Conflicts were resolved by a third party (DAC) when a consensus was not reached.

**Figure 1:**
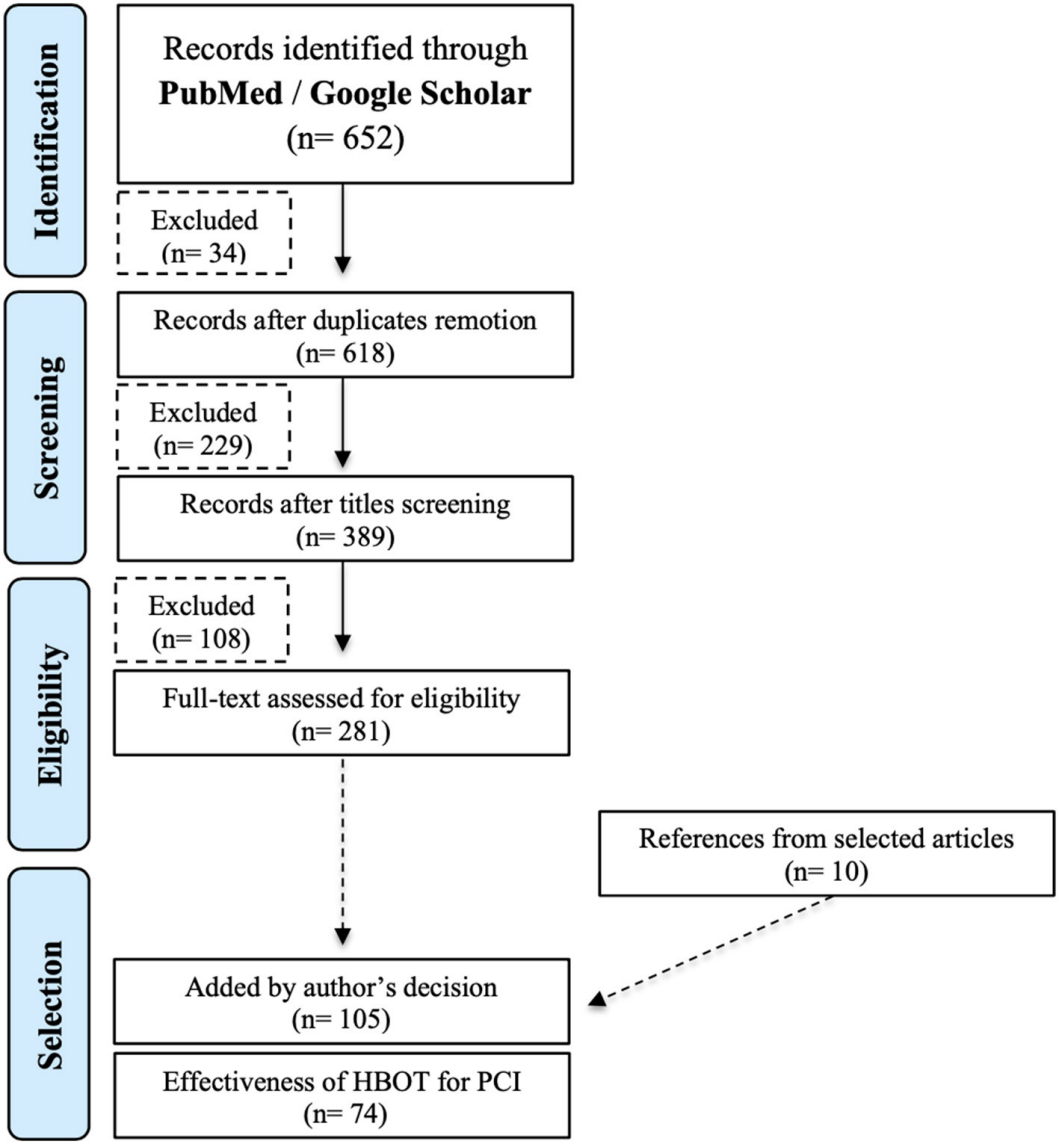
Flowchart explaining the article selection strategy (adapted from PRISMA, 2009). HBOT, hyperbaric oxygen therapy; PCI, pneumatosis cystoides intestinalis.

The initial search yielded 652 citations in PubMed and Google Scholar. Excluding duplicates, the number was reduced to 618. Title screen reduced the selection to 389 papers for reviewing abstracts, and 281 fulfilled the criteria for reading the full-text. References from selected articles were scanned in order to identify other 10 papers. In the final selection, 105 articles were chosen to be included in this review. Excluding literature reviews and duplication of data published in different articles, our assessment of the HBOT effectiveness for the treatment of PCI was based on 74 case series and case reports from 1978 to 2020 (Figure 1) (Supplementary Material).

We evaluated 89 patients with PCI treated with HBOT. Patient median age was 53 (0.8–98) years, of whom the majority 49 (55.1%) were female. Eighty-one percent (*n* = 73) had at least one underlying disease or risk factor for PCI. In this subgroup of patients, the predisposing factors were divided into different categories: pulmonary disorders (*n* = 10), mucosal disruption (*n* = 30), gastrointestinal motility disorders (*n* = 13), malignancies (*n* = 10), infectious (*n* = 5), immunological disturbances (*n* = 26), iatrogenic causes (*n* = 44) and intraabdominal complications (*n* = 3). In 18% of the patients (*n* = 16), the possible etiology for PCI was not discriminated in the previous past history and in some cases, it was even considered to be idiopathic. Almost half of the patients (*n* = 41; 45.5%) were treated previously or concurrently with other complementary treatment measures, including bowel rest, elemental diet, parenteral nutrition, antimotility agents, antacids, antimicrobials, anti-inflammatory drugs, normobaric oxygen and surgery. Administration of HBOT varied by study. Atmospheric pressure ranged from 1.9 to 3 atmosphere absolute (ATA), with a mean and median of 2.3 ATA and 2 ATA, respectively. Duration of exposures and number of HBOT sessions ranged from 40 to 150 min (min) and 1 to 80 treatment sessions, respectively. Beyond these protocols, six patients underwent treatment tables more adapted to decompression sickness [Comex 30 - heliox 50/50 (16) and US Navy Treatment Table 6 - USN TT6 and USN TT6A (17–19)]. In about 1/3 (*n* = 37) of the cases it was not possible to identify clearly the HBOT protocol regimen. Overall response rate (improvement or resolution of symptoms) was achieved in 92.1% (82 of 89) patients. Complete response (resolution of symptoms) was objectified in 65.2% (58 of 89) patients. There was one immediately complication related to HBOT (hearing disorder). (11) Other (adverse) events occurred during the peri-HBOT period (*n* = 9): death of an unknown cause (20), peripheral blood stem cell transplantation after (re)induction chemotherapy (21), one gastric cancer surgery (22) and six surgeries for complicated PCI (8, 23–27). Median follow-up was seven (0.5–120) months (2/3 of the patients lost to follow-up) (Table 1).

**Table 1:** Patient characteristics and outcomes from a critical literature review.

**Patient characteristics and outcomes**	
Number of patients	89
Age (mean/median)	50.6/53, yrs
Sex	49 (55.1%), female 36 (40.4%), male 4 (4.5%), unspecified
Underlying disease/Risk factor	Pulmonary disorders (*n* = 10): asthma (*n* = 5), chronic obstructive pulmonary disease (*n* = 2), interstitial pneumonia (*n* = 2), thymectomy (*n* =1) Mucosal disruption (*n* = 30): corrosive agents (*n* = 14), gastro-esophageal reflux disease/peptic ulcer disease (*n* = 6), inflammatory bowel disease (*n* = 4), endoscopic procedures (*n* = 2), prolapse rectal (*n* = 2), anorexia nervosa (*n* = 1), feeding jejunostomy (*n* = 1) Gastrointestinal motility disorders (*n* = 13): diabetes mellitus (*n* = 6), paralytic ileus (*n* = 2), achalasia (*n* = 1), chronic abdominal pain (*n* = 1), chronic constipation (*n* = 1), chronic idiopathic intestinal pseudo-obstruction (*n* = 1), gastroparesis (*n* = 1) Malignancies (*n* = 10): leukemia (*n* = 4), colon cancer (*n* = 2), cervix cancer (*n* = 1), gastric cancer (*n* = 1), head and neck cancer (*n* = 1), kidney cancer (*n* = 1) Infectious (*n* = 5): *Clostridium difficile* (*n* = 1), *Clostridium septicum* (*n* = 1), *Mycobacterium* spp. (*n* = 1), *Salmonella* spp. (*n* = 1), cytomegalovirus (*n* = 1) Immunological disturbances (*n* = 26): (rheumatoid) arthritis (*n* = 7), scleroderma (*n* = 5), polymyositis (*n* = 3), systemic lupus erythematosus (*n* = 3), Sjögren syndrome (*n* = 2), amyloidosis (*n* = 1), dermatomyositis (*n* = 1), granulomatosis with polyangiitis (Wegener granulomatosis) (*n* = 1), haemolytic anemia (*n* = 1), myasthenia gravis, Raynaud's phenomenon (*n* = 1) Iatrogenic causes (*n* = 44): steroids (*n* = 14), abdominal surgery (*n* = 10), other immunosuppressors (*n* = 7), chemotherapy agents (*n* = 4), NSAIDs (*n* = 3), organ transplantation (*n* = 3), targeted agents (*n* = 2), ionizing radiation (*n* = 1) Intraabdominal complications (*n* = 3): decompression sickness (*n* = 2), intussusception (*n*= 1)
Other (previous) treatments	Antimicrobials (*n* = 21) Bowel rest (*n* = 10) Antacids/Antimotility agents (*n* = 8) Normobaric oxygen (*n* = 7) Parenteral nutrition (*n* = 7) Anti-inflammatory drugs (*n* = 7) Elemental (personalized) diet (*n* = 4) Surgery (*n* = 1)
HBOT protocol (mean / median / min-max)	2.3/2.0 ATA/[40–150] min
Number of sessions (mean / median / min-max)	13.2/6 [1–80]
Overall response rate	92.1% (*n* = 82)
Complete response	65.2% (*n* = 58)
Follow-up period (median)	7 [0.5–120] mos
HBOT side effects	Hearing disorder (*n* = 1)
Other (adverse) outcomes	Intestinal surgery (*n* = 6) Gastric cancer surgery (*n* =1) PBSCT (*n* = 1) Death of an unknown cause (*n* = 1)

*ATA, atmosphere absolute; HBOT, hyperbaric oxygen therapy; Min, minute; Mos, months; NSAIDs, non-steroidal anti-inflammatory drugs; PBSCT, peripheral blood stem cell transplantation; Yrs, years*.

## Discussion

### Pathophysiology

**Figure 2:**
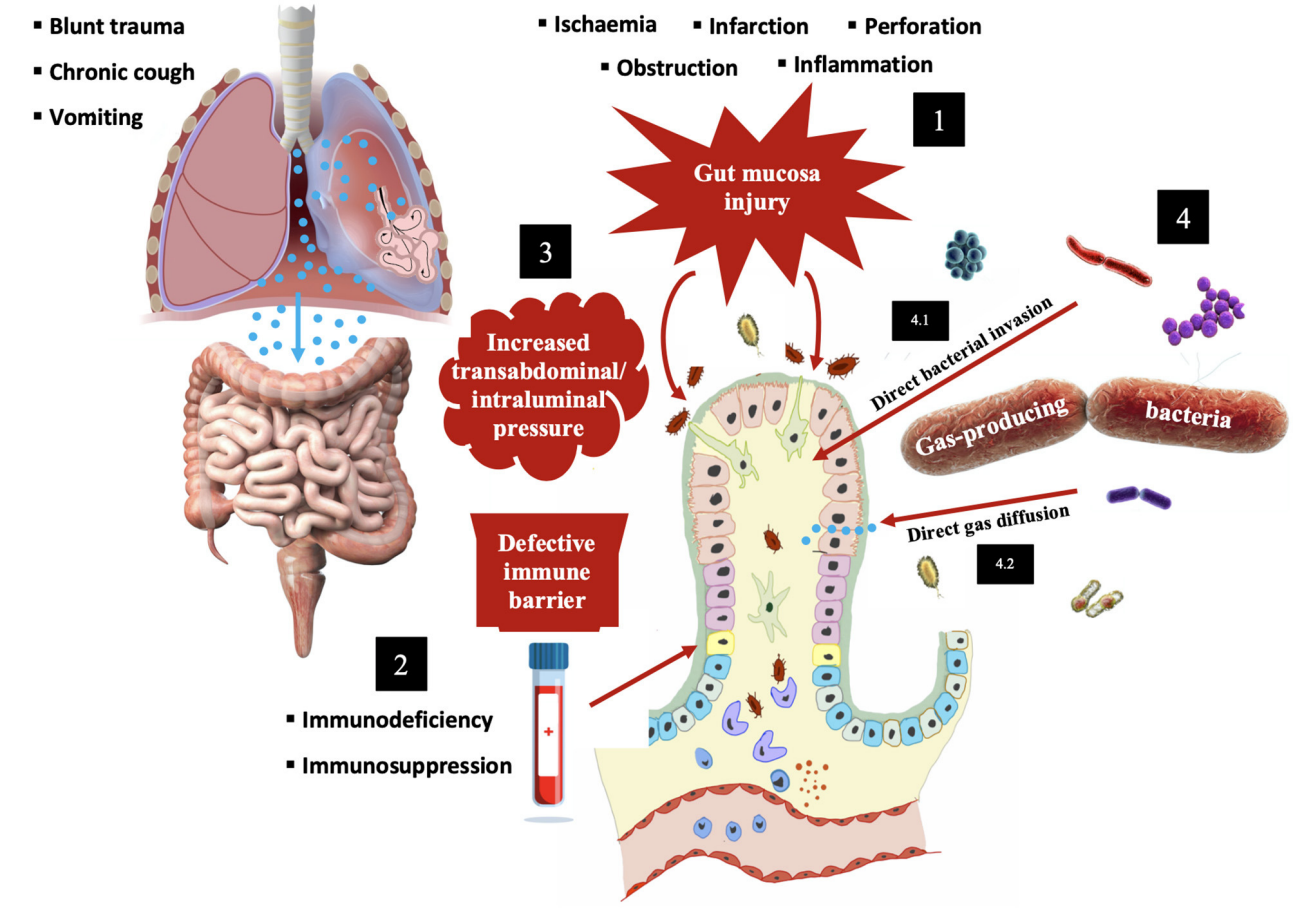
Schematic representation of the two most widely accepted theories for explaining PCI's development. Mechanical theory **(1–3)**. Migration of gas into the bowel wall through different routes: **(1)** Mucosal disruption with disturbance of normal continuity (inflammation, ulceration, trauma and necrosis); **(2)** Increased mucosal permeability due to defective immune barrier (immunodeficiency or immunosuppressive drugs); **(3)** Increased transabdominal/intraluminal pressure leading to direct gas diffusion into an intact mucosal barrier (bowel obstruction, chronic obstructive pulmonary disease, trauma or vomiting). The association of pulmonary disorders with PCI could be explained by the Macklin effect described in 1939 (28) and further studied by others (29). This pathophysiologic process consists in several steps: alveolar rupture with air dissecting along peribronchovascular interstitial sheaths and interlobular septa; spreading of this pulmonary emphysema into mediastinum; extension to retroperitoneum and mesenteric root, allowing air penetration across the bowel wall (4, 5, 8–11, 15, 16, 18, 28–30). Typically, a variable combination of these factors will contribute to gas dissection into intramural compartments. Bacterial theory **(4)**. There are two main complementary mechanisms to explain the contribution of bacterial microbiota for intramural gas production: **(4.1)** Gas-forming bacteria that directly penetrate gastrointestinal mucosa and submucosa (model tested and reproduced in laboratory with *Clostridium perfringens*). However, cysts appear to be sterile, since even after its rupture the pneumoperitoneum is not complicated by peritonitis; **(4.2)** Fermentation of bacterial gut microbiota converts carbohydrates and other nutrients into hydrogen gas. As the pressure of the intraluminal gas increases, there is a greater trend for it to migrate through the mucosa and become trapped in the submucosa. This phenomenon of gas gradient between intestinal lumen and blood that overcomes the bowel wall resistance is called “counterperfusion supersaturation.” Cysts can have as high as 50% hydrogen and, in some patients with PCI, the level of respiratory hydrogen is higher than in controls (4, 5, 8–11, 14–18, 21, 25, 27, 30–34).

The explanatory theories regarding PCI's pathogenesis are distinctly different in nature, but they also end up being intersected, with mechanisms involving increased transabdominal/intraluminal pressure, gut mucosal disruption, increased mucosal permeability and gas-producing bacteria. The current knowledge relies on two main theories – mechanical and bacterial (Figure 2).

### Clinical Presentation

Koss et al. (35) subcategorised PCI into primary/idiopathic (15%) or secondary (85%). PCI can be secondary to a wide of underlying conditions or predisposing factors: pulmonary disorders, mucosal disruption, gastrointestinal motility disorders, malignancies, infectious, immunological disturbances, iatrogenic causes and intraabdominal complications. Secondary PCI is traditionally divided into two categories: benign or non-urgent and life-threatening causes (Table 2) (4, 5, 8–11, 14–27, 29, 34–82).

**Table 2:** Causes of secondary pneumatosis cystoides intestinalis.

Pulmonary disorders	Asthma crisis, blunt or penetrating thoracic trauma, bronchiectasis, chronic obstructive pulmonary disease, cystic fibrosis, positive-pressure mechanical ventilation, pulmonary fibrosis, pulmonary tuberculosis, sarcoidosis, Valsalva manoeuvres (parturition, Boerhaave' syndrome, and epileptic seizures)
Mucosal disruption	Abdominal surgery, inflammatory bowel disease, corrosive agents, gastrointestinal endoscopy, ionising radiation, jejunostomy tubes, peptic ulcer disease, ruptured diverticulum, steroids
Gastrointestinal motility disorders	Chronic idiopathic intestinal pseudo-obstruction, chronic constipation, diabetes mellitus, gastroparesis Hirschsprung's disease, ileus, jejunoileal bypass, obstruction/pyloric stenosis, scleroderma
Malignancies	Gastrointestinal cancer, leukaemia and lymphoproliferative disorders
Infectious	AIDS-associated enterocolitis (*Cryptosporidium*, Cytomegalovirus*, Mycobacterium avium*), Adenovirus, Candida albicans, *Clostridium* spp., *Enterobacter* spp., *Klebsiella* spp., *Lactobacillus* spp., *Mycobacterium tuberculosis, Salmonella* spp., SARS-CoV-2, *Tropheryma whipplei*, Rotaviru*s*, varicella zoster virus
Immunological disturbances	AIDS, amyloidosis, bone marrow transplantation, corticosteroids, dermatomyositis, graft-versus-host disease, granulomatosis with polyangiitis (Wegener granulomatosis), mixed connective tissue disorder, polyarteritis nodosa, polymyositis, rheumatoid arthritis, sarcoidosis, scleroderma, Sjögren syndrome, solid organ transplantation, systemic lupus erythematosus
Iatrogenic causes	Abdominal surgery, antiacid medications, barium enema, bone marrow transplantation, bowel preparation, chemotherapy agents, chloral hydrate, gastrointestinal endoscopy, immunosuppressors, ionising radiation, jejunoileal bypass, jejunostomy tubes, lactulose, mechanical ventilation, nonsteroidal anti-inflammatory drugs, postsurgical anastomosis, sclerotherapy, steroids, solid organ transplantation, sorbitol, targeted therapy, α-glucosidase inhibitor
Intraabdominal complications	Intestinal ischaemia, infarction, intussusception, obstruction, perforation, mesenteric vascular disease, necrotising enterocolitis, toxic megacolon, blunt or penetrating abdominal trauma, decompression sickness

*AIDS, acquired immunodeficiency syndrome; SARS-CoV-2, severe acute respiratory syndrome coronavirus 2; Green color (benign/nonurgent)/Red color (life-threatening)*.

As previously mentioned, PCI can be diagnosed by chance in asymptomatic patients, while in others, it can be manifested with a plethora of constitutional and gastrointestinal symptoms. PCI-related symptoms are mainly related to the affected gastrointestinal tract region and the underlying condition that led to its onset. In small intestine, the most frequent symptoms are nausea, vomiting, abdominal bloating and pain, weight loss and diarrhea and, in relation to colonic pneumatosis, the symptoms are abdominal bloating and pain, constipation, flatulence, diarrhea, tenesmus and haematochezia. Intraabdominal complications of PCI occur in <5% of patients and include bowel obstruction driven by the cyst *per se*, intussusception, volvulus, adhesions or pneumoperitoneum after air-filled cyst's rupture, haematochezia due to mucosa ulceration, and intestinal ischaemia (4, 5, 8–11, 13, 34, 69, 83).

**Figure 3:**
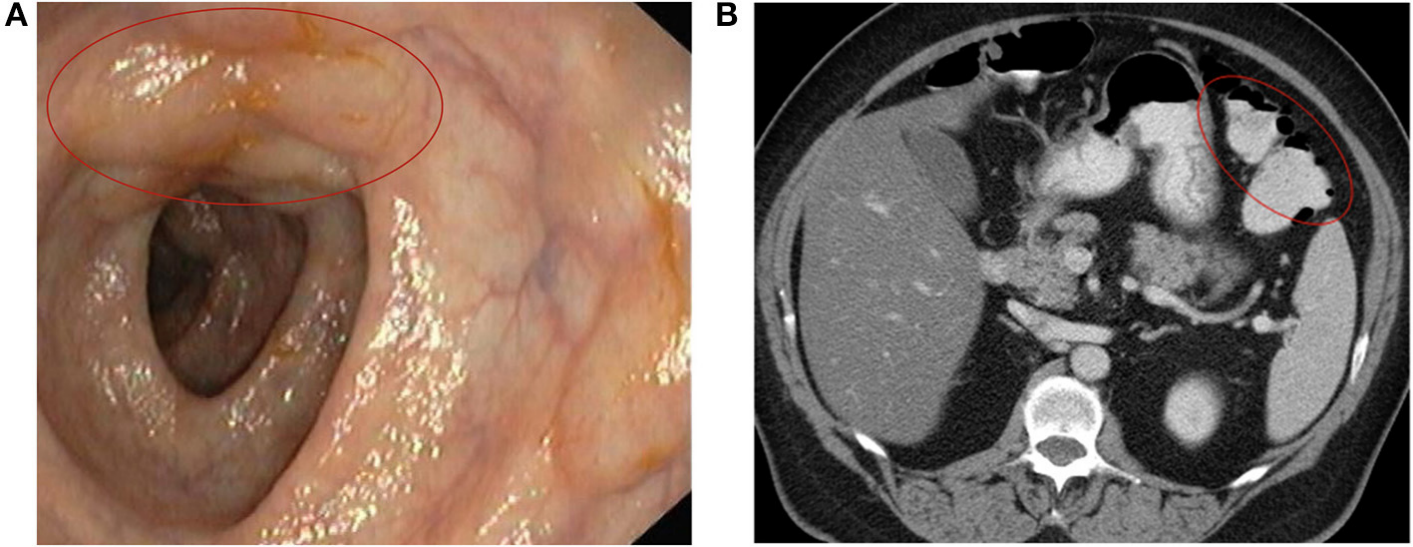
Pretreatment images of a 59-year-old woman with PCI that underwent HBOT at our centre (69) **(A)** PCI's endoscopic image of subepithelial nodular elevations covered with mucosa of normal appearance **(B)** Abdominal computed tomography showing gas-filled cysts in the splenic flexure of the colon.

PCI is usually detected on imaging or endoscopy performed for evaluation of abdominal symptoms. The radiological findings may be present on several imaging modalities. However, computed tomography scan is more sensitive than plain radiography, indicating a potential underlying cause and a better distinction between an indolent and complicated PCI, such as bowel wall thickening, dilated bowel, vessels occlusion, hepatic portal or portomesenteric venous gas, ascites, etc. (Figure 3) (5, 9, 10, 15, 18, 19, 26, 48, 57, 59, 60, 62, 66, 69, 79, 80).

### Treatment

PCI may disappear spontaneously, persist for many years, or relapse after treatment. However, there are no consensus guidelines on PCI's management. The therapeutic approach reflects the (still) scarce knowledge of its pathophysiology, ranging from conservative treatment in mild and moderate cases to exploratory laparotomy in highly symptomatic patients with intraabdominal complications (4, 5, 9, 10, 13, 30, 34, 69, 83). Furthermore, a potentially reversible PCI's secondary cause must also be ruled out and treated (e.g., targeted therapy with axitinib) (73).

Asymptomatic patients do not require any complementary treatment (5, 54). Patients with mild symptoms can be managed on an outpatient basis with a combination of antibiotics cycles and an elemental diet (5, 34). Sclerotherapy has also been used with success (4, 69). For patients with moderate to severe symptoms, a combination of antibiotics, an elemental diet and HBOT is suggested (5, 26, 42, 69, 83). Patients with refractory symptoms and intraabdominal complications have an indication for surgery approach (5, 18, 23–27, 69). Clinical and radiological follow-up should be performed based on each case-based analysis.

### Hyperbaric Oxygen Therapy

HBOT is a treatment based on the inhalation of pure oxygen (100%) in an environment with atmospheric pressure higher than that existing at sea level (1 ATA). The HBOT sessions are held inside hermetically sealed hyperbaric chambers, which are classified as type IIb medical devices (Directive 93/42 ECC of June 14, 1993, concerning medical devices). HBOT is used in a number of clinical conditions as well as in professional and military training. In clinical practice, HBOT usually involves pressures higher than 1.4 ATA, frequently ranging between 2.0 and 2.5 ATA for 90 to 120 min (84).

Both normobaric and HBOT have been used to treat PCI not responding to conservative measures. The rationale behind the beneficial action of oxygen is related to two main physiological effects, addressing some of the aforementioned pathophysiological mechanisms of PCI:

- ■ The reduction in inert gases volume and diameter provided by ambient pressure elevation (Boyle's law), creates a pressure gradient which promotes oxygen diffusion into the gas-filled cysts, while hydrogen and nitrogen moves out. Hence, HBOT may restore normal intestinal motility by removing luminal and bowel wall gas. This mechanism is enhanced by HBOT in relation to normobaric oxygen, given the greater diffusion gradients generated by oxygen administered under pressure (> 2 ATA) (5, 14, 17, 18, 34, 42, 69, 85, 86).
- ■ Oxygen contributes to antimicrobial activity, especially for obligate and facultative anaerobic gas-producing bacteria, promoting bacteriostatic/bactericidal effects, leukocyte diapedesis and phagocytosis. In addition, HBOT potentiates the action of some antibiotics, increasing their local concentration, which is why the combination of these treatments makes sense in PCI' management (5, 15, 34, 44, 71, 87, 88).

The use of high concentrations of oxygen in the treatment of gas containing cavities was first proposed in 1935 (89). However, it was only in 1973 by Forgacs et al. (85) that oxygen inhalation was successfully used in PCI' treatment. At that time, the therapeutic protocol comprised an intensive regimen with normobaric oxygen therapy (5 h/day, 7 days) to achieve a PO_2_ of 200 to 300 mmHg. The drawback of this protocol was related to the increased risk of pulmonary toxicity (11, 20, 34, 44, 48, 56, 85).

In the late 70s, Masterson et al. (14) used HBOT (2.5 ATA, 120 min, 2 or 3 consecutive days) with a clinically significant benefit in 2 patients with PCI. Since then, several case reports, case series, and reviews have been published in the literature. In 1991, Grieve and Unsworth (42) reported a series with 8 patients treated with HBOT. As there was no standard regimen, the number, pressure and duration of treatment sessions were variable (ranged, 6–11, 1.9–2.8 ATA and 70–135 min, respectively). With the exception of 2 patients, treatment sessions were twice daily. All patients responded to treatment, with 4 long-term complete remissions. Two patients died of another cause. In 2001, Shimada et al. (48), in a Japanese literature review, reported the outcomes of 15 patients with PCI treated with HBOT (2 to 3 ATA, 60 to 120 min/day, ranging from 3 to 33 days). PCI resolved in 11 patients, improved in the other 4, and relapsed in 1 patient 9 months after treatment. There were no serious adverse events registered. In 2004, in another Japanese literature review, by Togawa et al. (54), outcomes were directly compared among two groups of patients with PCI. Seven patients underwent HBOT and 20 patients to normobaric oxygen with a number of treatment days of 1–8 (mean, 4.7) and 1–35 (mean, 14.6), respectively. Despite the bias of a direct comparison, a shorter treatment period required for HBOT patients may suggest a better therapeutic effect. In 2005, Tahiri et al. (90), proposed a different treatment approach based on decision-making factors, such as clinical, laboratorial and imaging data, considering HBOT in the absence of surgery indication. More recently, in 2014, in the largest literature review published so far, by Feuerstein JD et al. (5), 35 illustrative cases of PCI treated with HBOT were identified. Symptomatic resolution or improvement was achieved in 89% of patients (31/35). Although, the therapeutic protocol was not uniform across the case reports. These authors proposed a treatment regimen of HBOT at 2.5 ATA during 120 min for at least 3 sessions in patients with symptomatic and non-emergent PCI. After this Mayo Clinic concise review (5), more case reports were published in the literature (Supplementary Material) (5, 11, 14–27, 34, 36–81, 88, 91–101).

To the best of our knowledge, we included in our scoping review the important studies regarding the effectiveness of HBOT for PCI in the “real world” clinical practice. We considered 74 studies comprising 89 patients with PCI treated with HBOT, including one patient of our center. In this 42-year analysis the overall response rate and complete response were 92.1 and 65.2%, respectively, for a median follow-up of 7 (0.5–120) months. These numbers reinforce the data assessment reported in previous literature reviews. It should also be noted that almost half of the patients were previously treated or concurrently with other complementary treatments, evidencing the importance of a multimodal approach of this gastrointestinal pathology. However, a long-term follow-up evaluation is suggested to determine the long-term outcome of HBOT and other treatments in this setting.

Interestingly, the men-to-women ratio was not what would be predictable according to what was published in one of the largest population-based systematic reviews with 239 PCI cases (11). On the contrary, the age of our cohort was in line with what was published in the same study (11). These facts are probably more related to the small sample size and to the possible predisposing conditions for PCI, including, for example, the immunological disturbances that are generally more frequent in females (102). In this review, 28.9% of patients (26/90) had a background autoimmune disease, and the majority were female (only 2 males).

Despite the very favorable data on clinical benefit of HBOT for PCI, it is difficult to compare the protocols that have used different hyperbaric protocols regimens (PO_2_ pressures, air intervals, duration of exposures and number of HBOT sessions), various techniques for oxygen delivery (hood, mask, monoplace) and several (or absent) complementary treatments that were variable among the studies. In addition, differences in the type of patients included in this review with different ages, comorbidities and clinical conditions also contribute to further consolidating this heterogeneity. The ATA level, duration and number of HBOT sessions ranged from 1.9 to 3 ATA, 40 to 150 min and 1 to 80 treatment sessions, respectively. Different HBOT protocols (Comex 30 – heliox 50/50 and USN TT 6/TT6A) were performed in six patients (16–19). These types of treatment regimens that are better clinical validated for severe decompression sickness and gas embolism, were used in three cases of hydrogen peroxide ingestion (17) and in another case with no apparent underlying cause for PCI, besides chronic constipation (16). The complete response rate was 100% in all these cases, with the exception of one of the cases of severe decompression illness with mesenteric venous thrombosis and PCI (18). Despite the initial mitigation of intravenous gas according to the image findings, the clinical course worsened with the need for surgery and admission to the intensive care unit (18). To avoid this outcome, could the treatment have been intensified with increased pressure and duration of exposure? Hence, bubbles of inert gas would be eliminated more quickly. In the other case of severe decompression sickness, the compression was upgraded to 6.0 ATA to fit the USN TT6A with a clear improvement of symptoms (19). Probably in critically ill patients with PCI following decompression sickness with no significant improvement 30 min after recompression at 2.8 ATA, treatment should be further intensified.

There was one immediately complication related to HBOT (hearing disorder) (11). Probably it was a tympanic barotrauma, the most frequent HBOT side effect (103). During the peri-HBOT period, we should highlight a death of an unknown cause (20), a peripheral blood stem cell transplantation (21), 1 gastric cancer surgery (22) and 6 surgeries for complicated PCI. (8, 23–27) The patient who died had undergone HBOT a few days before with improvement of PCI. However, shortly thereafter, the patient worsened dramatically with pleural effusion, haemoptysis, severe respiratory failure, and eventually died (20). Given that an autopsy was not performed, it was not possible to determine the cause of death. We can speculate that this fatal outcome could have been related to the progression of head and neck cancer with lung metastases or even, although being a more remote hypothesis, with a pulmonary complication of HBOT. In the case related to bone marrow transplantation, the patient developed PCI after re-induction chemotherapy in the context of an acute myeloid leukemia. The combination of HBOT with broad-spectrum antimicrobial agents enabled the resolution of PCI, avoiding delays that could impact the ideal timing for transplantation (21). Interestingly, during the gastric cancer surgery, it was witnessed the live response to HBOT in the sigmoid and transverse colon with PCI. After 1 month, PCI complete remission was observed (22). Six patients with slight improvement or refractory symptoms to HBOT underwent intestinal surgery.

Notwithstanding the clinical value of HBOT, when PCI is secondary to gas entry *via* pulmonary disorders, there may be a worsening of the clinical picture with intestinal expansion during decompression. Thus, HBOT should be performed carefully in these specific settings.

The current analysis is limited by several factors, including its retrospective nature, evidence based on case reports, population heterogeneity and incomplete registration data in relation to the patient's characteristics/outcomes and to the clinical context of PCI and HBOT protocols. Nevertheless, the authors believe that the current findings represent an accurate depiction of the clinical effectiveness of HBOT for PCI, including in severe clinical conditions underrepresented in other literature reviews.

With the growing requirement for evidence-based research, HBOT has been criticized for delivering too little high-quality research, mainly in the form of randomized controlled trials. Nevertheless, the design of a sham (hyperbaric) treatment is associated with considerations regarding adequate blinding and the use of pressure and oxygen (104). Additionally, considering the rarity of PCI and the logistical issues of HBOT, it is difficult to admit that in the future it will be possible to conduct randomized controlled clinical trials in this particular context. Currently, HBOT is considered by the European Committee for Hyperbaric Medicine (ECHM) as a modality of treatment for PCI (degree of recommendation II/level of evidence C) (105).

**Figure 4:**
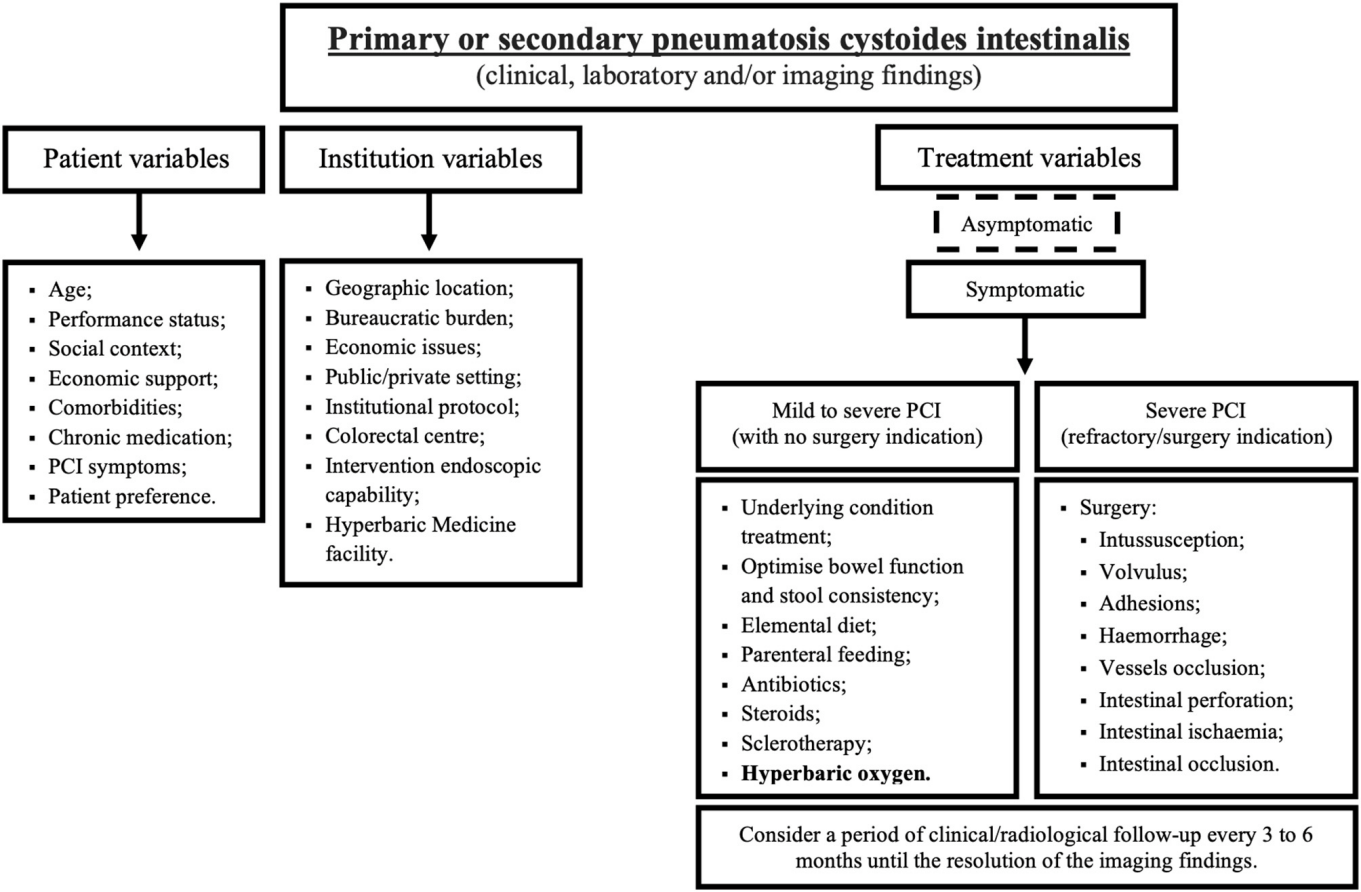
Suggested treatment algorithm for pneumatosis cystoides intestinalis. PCI, pneumatosis cystoides intestinalis.

We recommend that HBOT, in combination with elemental diet and antibiotics, may be beneficial for patients with moderate to severe symptoms and not requiring emergent exploratory laparotomy. The HBOT regimen should include at least 3 sessions of 2 to 3 ATA and between 60 to 120 min/day to ensure a more effective clinical response. The total duration of the treatment protocol could be extended to several weeks until clinical and radiological response and the follow-up should be personalized to each clinical context. We suggest that radiological follow-up be performed after symptoms control or every 3 to 6 months until the resolution of the imaging findings (Figure 4).

## Conclusions

Herein, we reported the relevant literature review on PCI's epidemiology, pathophysiology, clinical presentation and analyzing the results of previous HBOT studies and discussing PCI' management, including the proposal of an algorithm, which takes into account the variables related to the patient, institution, and clinical context severity.

Although the identification of the underlying cause of PCI is crucial for the prognosis and treatment, it is not well-defined how or if it affects HBOT's response. HBOT probably addresses some of the underlying PCI's pathophysiological mechanisms. There were an overall response rate and complete response rate of 92.1 and 65.2%, respectively, for a median follow-up of 7 months. The optimal concentration, duration, and effect of oxygen have not yet been precisely determined. A long-term follow-up evaluation is suggested to determine the long-term outcome of HBOT and other treatments in this setting.

There is a lack of randomized or prospective data on the application of HBOT for PCI, and this would normally limit the strength of recommendations for its use. A randomized, controlled, and double-blind clinical trial is unlikely to occur given the rarity of PCI, the logistical issues and the several methodological considerations regarding adequate blinding with the addition of a sham-controlled group. Although, despite the absence of any high-level evidence, the resolution of symptoms in most retrospective studies with HBOT may support recommendations for its use as a treatment strategy for PCI. Therefore, the publication of the experience from different Hyperbaric Medicine Centers in PCI's management is essential to better validate its effectiveness in the “real world” clinical practice.

## Consent for Publication

All authors have given their permission for publishing the manuscript, have read the submission and agree to be listed as co-authors.

## Author Contributions

DA: conception and design, acquisition, analysis, and interpretation of data. DA and PM: writing, review, and/or revision of the manuscript. JV: manuscript supervision. The present manuscript is the result of original work by all the authors.

## Conflict of Interest

The authors declare that the research was conducted in the absence of any commercial or financial relationships that could be construed as a potential conflict of interest.
