# Supplementary material for: The Role of Hyperbaric Oxygen Therapy in Pneumatosis Cystoides Intestinalis—A Scoping Review
Source: Front Med (Lausanne). 2021 Feb 17;8:601872. doi: 10.3389/fmed.2021.601872 (PMC7926085; doi:10.3389/fmed.2021.601872)
Supplement: Supplementary file 1 [file Data_Sheet_1.PDF]

SUPPLEMENTARY MATERIAL

Summary of a critical literature (1978-2020) for pneumatosis cystoides intestinalis treated with hyperbaric oxygen therapy.

First author[ref.] (year)	Study design	Pts. (Nº)	Age [min-max] (years) / Sex Nº (M / F)	Underlying disease / Risk factor	Other (previous) treatments (%)	HBOT protocol	Sessions (Nº) [min-max]	Overall response (%) (Y/N)	CR (%) (Y/N)	FU period [min-max] (months)
**Masterson *et al.*[14] (1978)**	Case series	2	86 / F<br>56 / F	1st case: asthma, hysterectomy, partial thyroidectomy<br>2nd case: idiopathic	No	2.5 ATA, [120-150] min, consecutive days	1st case: 2<br>2nd case: 3	100	50	[2.5-NA]
**Tsiftsis *et al.*[91] (1979)**	Case report	1	58 / F	Idiopathic	No	NA	NA	Yes	No	24
**Daitoku *et al.*[92] (1980)**	Case report	1	60 / M	NA	NA	2.5 ATA, 120 min, 3 days	NA	Yes	No	NA
**Ellis et al.[88] (1983)**	Case series	2	NA	NA	NA	2.0 ATA, 120 min alternating with 240 min out of hyperbaric chamber; maximum of 14 hours of treatment	NA	50	NA	NA
**Kobayashi *et al.*[36] (1983)**	Case report	1	63 / F	Asthma	NA	2.8 ATA, 90 min, 15 days	NA	Yes	Yes	NA
**Ohnishi *et al.*[37] (1984)**	Case report	1	47 / M	Asthma, colon cancer	NA	2.1 ATA, 50 min, 24 days	NA	Yes	Yes	NA
**Park *et al.*[38] (1985)**	Case report	1	76 / F	Polymyositis	NA	[2.0-3.0] ATA, [60-90] min, 33 days	NA	Yes	No	NA
**Goto *et al.*[93] (1986)**	Case report	1	72 / M	NA	NA	2.0 ATA, 120 min, 15 days	NA	Yes	No	9
**Hara *et al.*[94] (1988)**	Case report	1	58 / F	NA	NA	2.0 ATA, 60 min, 10 days	NA	Yes	Yes	NA
**Honda *et al.*[39] (1988)**	Case report	1	29 / F	Scleroderma	NA	NA	NA	Yes	Yes	NA
**Ogata *et al.*[40] (1988)**	Case report	1	23 / M	Trichloroethylene exposure, appendectomy	No	O_2 50%, ATA/duration NA, daily	10	Yes	Yes	NA
**Yokoi *et al.*[41] (1989)**	Case report	1	50 / M	Trichloroethylene exposure	NA	3.0 ATA, 150 min, 6 days	NA	Yes	Yes	NA
**Grieve *et al.*[42] (1991)**	Case series	8	[45-85]	1st case: hyperthyroidism<br>2nd case: arthritis, bronchitis, NSAIDs	Normobaric oxygen, antibiotics	[1.9-2.8] ATA, [70-135] min,	[6-11]	100	50	120

			/<br><br>7 (F)<br>1 (M)	3rd case: hypothyroidism, arthritis, systemic lupus erythematosus; NSAIDs<br>4th case: hypothyroidism<br>5th case: asthma<br>6th case: peptic ulcer disease, rectal prolapse<br>7th case: hypothyroidism, arthritis<br>8th case: emphysema		once or twice daily				
**Yoshida *et al.*[95] (1991)**	Case report	1	51 / F	NA	No	2.5 ATA, 60 min, daily	8	Yes	Yes	48
**Carli *et al.*[43] (1992)**	Case report	1	50 / F	Rheumatoid arthritis, rheumatoid pericardial disease, Sjögren syndrome; methotrexate, steroids	Metronidazole	NA	NA	Yes	Yes	0.5
**Ina *et al.*[44] (1993)**	Case report	2	52 / M<br>61 / M	1st case: idiopathic<br>2nd case: asthma, trichloroethylene exposure	NA	2.0 ATA, 75 min, once or twice daily	1st case: 10<br>2nd case: 30	100	100	[11-21]
**Iitsuka *et al.*[45] (1993)**	Case report	1	28 / F	Nephrotic syndrome; steroids	Normobaric oxygen	NA	NA	Yes	NA	NA
**Kohzaki *et al.*[20] (1994)**	Case report	1	51 / M	Head and neck cancer with lung metastases; chemoradiotherapy, steroids	NA	NA	NA	Yes	No	Died
**Kishimoto *et al.*[21] (1995)**	Case report	1	72 / M	Peptic ulcer disease, gastrectomy (Billroth I) 28 yrs before (gastric cancer)	NA	2.5 ATA, 120 min	3	Yes	No	Gastric cancer
**Satoh *et al.*[46] (1995)**	Case report	1	35 / F	Scleroderma	Normobaric oxygen, parenteral nutrition	NA	8	Yes	Yes	NA
**Paw et al.[96] (1996)**	Case report	1	72 / M	Idiopathic	Bowel rest, parenteral nutrition	2.0 ATA, 60 min (10 sessions in 2 wks. -> 4 sessions in 2 wks. -> 4 sessions in 4 wks.)	18	100	Yes	3
**Oka et al.[97] (1999)**	Case report	1	58 / F	Idiopathic	No	2.0 ATA, 60 min, daily	5	100	Yes	6
**Fujioka *et al.*[47] (2000)**	Case report	1	57 / F	Trichloroethylene exposure, atrial septal defect surgery	Normobaric oxygen	2.0 ATA, 120 min, NA	NA	100	Yes	NA
**Limura et al.[98] (2000)**	Case report	1	29 / F	Idiopathic	Bowel rest, cisapride, erythromycin	2.0 ATA, 60 min, NA	18	100	No	NA
**Stollman *et al.*[99] (2000)**	Case report	1	70 / M	Idiopathic	NA	NA	NA	No	-	NA
**Shimada *et al.*[48] (2001)**	Case report<br><br>and<br><br>Japanese literature review	15	[23-76]<br><br>/<br><br>8 (F)<br>7 (M)	1st case: NA<br>2nd case: asthma<br>3rd case: asthma, colon cancer<br>4th case: polymyositis<br>5th case: NA<br>6th case: NA<br>7th case: NA<br>8th case: systemic lupus erythematosus<br>9th case: trichloroethylene exposure	NA	[2.0-3.0] ATA, [60-150] min, once or twice daily, [3-33] days	[3-60]	100	73	NA

	(1980-2000)			10th case: trichloroethylene exposure<br>11th case: NA<br>12th case: asthma, trichloroethylene exposure<br>13th case: scleroderma<br>14th case: chronic idiopathic intestinal pseudo-obstruction<br>15th case: Sjögren syndrome						
**Imashuku *et al.*[22] (2002)**	Case report	1	NA	Acute myeloid leukaemia; induction and re-induction chemotherapy	Amphotericin B, polymyxin B and vancomycin	NA	NA	Yes	Yes	PBSCT
**Lustberg *et al.*[49] (2002)**	Case report	1	64 / F	Diabetes mellitus, osteoarthritis; NSAIDs	Mesalamine, antibiotics, antimotility agents	2.0 ATA, 90 min (10 sessions -> 14 sessions -> 4 sessions in 2 wks. -> 4 sessions in 4 wks.)	32	Yes	Yes	12
**Machida *et al.*[50] (2002)**	Case report	1	76 / M	Several abdominopelvic surgeries	Emergency laparotomy	NA	3	Yes	Yes	9
**Qureshi *et al.*[51] (2002)**	Case report	1	69 / M	Diabetes mellitus	Dietary changes, antidiarrheals, antacids	2.4 ATA, 110 min	3	Yes	Yes	6
**Takada *et al.*[52] (2002)**	Case report	1	10 mos / F	Leukaemia; pirarubicin hydrochloride, aclarubicin hydrochloride, etoposide, cytarabine	Antibiotics, antifungal drugs	NA	NA	Yes	Yes	NA
**Tomiyama *et al.*[53] (2003)**	Case report	1	77 / M	Diabetes mellitus	Normobaric oxygen	2.8 ATA, 60 min (3 sessions) -> 2.5 ATA, 60 min (7 sessions), daily	10	Yes	Yes	NA
**Togawa *et al.*[54] (2004)**	Case series	2	38 / F<br>53 / F	1st case: myasthenia gravis, thymectomy; steroids<br>2nd case: scleroderma, polymyositis, rheumatoid arthritis; steroids	Steroids	1st case: 2.0 ATA, 60 min (9 sessions) -> 2.8 ATA, 120 min (12 sessions)<br>2nd case: 2.0 ATA, 60 min (5 sessions)	1st case: 21<br>2nd case: 5	100	50	NA
**Togawa *et al.*[54] (2004)**	Japanese literature review (1991-2000)	7	NA	Scleroderma, chronic idiopathic intestinal pseudo-obstruction	NA	[2.0-3.0] ATA, [60-NA] min, variable schedule	Variable	100	100	NA
**Lomb *et al.*[55] (2005)**	Case report	1	64 / M	Coeliac disease	Parenteral nutrition, special gliadin free diet, metronidazole, steroids	NA	NA	Yes	Yes	12
**Yoshizawa *et al.*[56] (2005)**	Case report	1	60 / M	Trichloroethylene exposure, lung tuberculosis	No	2.0 ATA, 60 min, daily, 20 days -> 2.0 ATA, 60 min, twice daily, 30 days	80	Yes	Yes	12
**Donati *et al.*[57] (2007)**	Case report	1	32 / F	Coeliac disease, *Salmonella* spp.	Antibiotics	NA	NA	Yes	Yes	1

**Yoshizawa *et al.*[58] (2007)**	Case report	1	18 / F	Anorexia nervosa (self-induced vomiting and constipation)	NA	2.0 ATA, [85-170] min, 5 times per wk.	24	Yes	Yes	0.5
**Hermsen *et al.*[59] (2008)**	Case report	1	5 / F	Acute lymphocytic leukaemia; vincristine and PEG-asparaginase, *Clostridium septicum*	Vancomycin, imipenem, ceftriaxone, cefoxitin, clindamycin, penicillin G	NA (3 wks.)	NA	Yes	Yes	8
**Ito *et al.*[60] (2008)**	Case report	1	45 / F	Chronic toluene inhalation	NA	2.0 ATA, 90 min	1	Yes	Yes	1.5
**Mizoguchi *et al.*[61] (2008)**	Case report	1	35 / F	Systemic lupus erythematosus, pleuritis, pericarditis, haemolytic anaemia; tacrolimus, cyclophosphamide, steroids	Bowel rest, parenteral nutrition, kanamycin, octreotide, metoclopramide, mosapride, erythromycin, steroids	NA	NA	Yes	NA	2
**Hokama *et al.*[62] (2009)**	Case report	1	56 / F	Rheumatoid arthritis	NA	NA	NA	Yes	Yes	NA
**Chaput *et al.*[100] (2010)**	Case report	1	57 / M	Idiopathic	No	2.5 ATA, 60 min, twice daily	50	Yes	Yes	6
**Fushimi *et al.*[63] (2010)**	Case report	1	51 / M	Polymyositis, interstitial pneumonia; cyclosporine, methylprednisolone	Normobaric oxygen, bowel rest	ATA NA,60 min, daily	NA	Yes	Yes	24
**Azzaroli *et al.*[64] (2011)**	Case report	1	44 / NA	Coeliac disease	Bowel rest, antibiotics	2.5 ATA, 75 min	5	Yes	Yes	NA
**Frossard *et al.*[16] (2011)**	Case report	1	56 / M	Chronic constipation	Personalised diet, bowel transit moderator, antibiotics, painkillers	Comex 30 (heliox 50/50) (2 sessions) -> 2.5 ATA, 90 min (1 session) -> Comex 30 (1 session) -> 2.5 ATA, 90 min (13 sessions)	17	Yes	Yes	6
**Kwon *et al.*[65] (2011)**	Case report	1	33 / F	Hepatitis B virus-related cirrhosis who underwent living donor liver transplantation; FK 506, mycophenolic mofetil, prednisolone	NA	NA	NA	Yes	Yes	NA
**Nakano *et al.*[23] (2011)**	Case report	1	31 / F	Ulcerative colitis, steroids	NA	NA	NA	Yes	No	Surgery
**Hokama *et al.*[66] (2012)**	Case report	1	23 / F	Systemic lupus erythematosus	NA	NA	NA	Yes	NA	NA
**Wu *et al.*[11] (2013)**	Case report	1	70 / M	Diabetes mellitus	Antibiotics	NA	NA	NA	-	HBOT side effect (hearing disorder)
**Feuerstein *et al.*[5] (2014)**	Literature review (1978-2010)	35	[10 mos - 86 yrs]	Pulmonary disorders: asthma, chronic obstructive pulmonary disease	NA	[1.9-3.0] ATA, 60-120 min, variable schedule	[1-60]	89	NA	NA

			/ NA (F / M)	Mucosal disruption: corrosive agents, inflammatory bowel disease, peptic ulcer disease, steroids<br>Gastrointestinal motility disorders: chronic idiopathic intestinal pseudo-obstruction, chronic constipation, diabetes mellitus<br>Malignancies: colon cancer, leukaemia<br>Immunological disturbances: polymyositis, rheumatoid arthritis, scleroderma, Sjögren syndrome, systemic lupus erythematosus<br>Iatrogenic causes: abdominal surgery, chemotherapy, NSAIDs, steroids						
**Gotoh *et al.*[24] (2014)**	Case report	1	17 / M	Intussusception	NA	NA	NA	No	-	Surgery
**Inoue et al.[67] (2014)**	Case report	1	83 / F	Idiopathic	NA	2.0 ATA, 60 min	7	Yes	Yes	NA
**Anonymous[68] (2015)**	Case report	1	30 / F	Hydrogen peroxide ingestion	NA	NA	1	Yes	Yes	NA
**Costa *et al.*[69] (2015)**	Case report	1	59 / F	Diabetes mellitus, total hysterectomy with bilateral adnexectomy	Metronidazole	2.5 ATA, 75 min, 5x/wk.	80	Yes	Yes	NA
**Garofalo *et al.*[70] (2015)**	Case report	1	4 / F	Heart transplantation; antithymocyte globulin, mycophenolate mofetil, rituximab, tacrolimus, prednisone	Bowell rest, parenteral nutrition, vancomycin, meropenem, steroids	NA	14	Yes	Yes	1
**Girardin *et al.*[71] (2015)**	Case report	1	50 / F	Heart transplantation for refractory heart failure due to amyloidosis with cardiac involvement, *Clostridium difficile*, cytomegalovirus; cyclosporine, human immunoglobulin, steroids	Metronidazole, ciprofloxacin, vancomycin, ganciclovir	2.5 ATA, 90 min, daily	14	Yes	Yes	1
**Prakash *et al.*[72] (2015)**	Case report	1	29 / F	Scleroderma, gastroparesis, chronic abdominal pain	Bowel rest, total parenteral nutrition, antibiotics	NA	3	Yes	Yes	NA
**Yagisawa *et al.*[73] (2015)**	Case report	1	67 / M	Kidney cancer with lung metastases and suspected duodenum and colon invasion, radical nephrectomy; temsirolimus, axitinib	Temporary axitinib discontinuation	NA	NA	Yes	Yes	Axitinib resumed
**Bang *et al.*[74] (2016)**	Case report	1	55 / M	Corrosive ingestion, feeding jejunostomy, endoscopic dilatation of oesophagus	NA	2.0 ATA, 90 min, daily	11	Yes	No	NA
**Castren *et al.*[15] (2016)**	Case report	1	74 / F	Cervix cancer treated with chemoradiotherapy and brachytherapy, colon adenocarcinoma, gastro-oesophageal reflux disease, achalasia recently managed with gastroscopy and botulinum toxin injections, abdominal surgery	Bowel rest, total parenteral nutrition, antibiotics	NA	1	Yes	Yes	1.5
**Fujimi *et al.*[75] (2016)**	Case report	1	55 / M	Idiopathic interstitial pneumonia, myocardial infarction, chronic heart failure, paroxysmal atrial fibrillation, gastro-oesophageal reflux disease,	Prokinetic agents, antibiotics	NA	5	Yes	Yes	18

				constipation, history of paralytic ileus, chronic myelogenous leukaemia in the chronic phase; nilotinib, steroids						
**Miyamae *et al.*[25] (2016)**	Case report	1	16 / F	Dermatomyositis, steroids	Bowel rest, steroids	NA	NA	Yes	No	Surgery
**Tong *et al.*[101] (2016)**	Case series	2	34 / F<br>38 / M	Idiopathic	NA	NA	NA	100	NA	NA
**Calabrese *et al.*[34] (2017)**	Case report	1	Late 60s / F	Scleroderma, Raynaud's phenomenon, diabetes mellitus, gastro-oesophageal reflux disease, chronic rectal prolapse repaired by complete proctectomy and colostomy	Bowel rest, metronidazole, methylprednisolone	2.0 ATA, 120 min, daily	3	Yes	NA	NA
**Hendriksen *et al.*[17] (2017)**	Case series	4	[33-98]<br>/<br>2 (F)<br>2 (M)	Hydrogen peroxide ingestion	NA	1st case: 2.8 ATA, 40 min<br>2nd case: USN TT6** (2 sessions)<br>3rd-4th cases: USN TT6** (1 session)	[1-2]	100	100	NA
**Martin *et al.*[76] (2017)**	Case report	1	51 / F	Hydrogen peroxide ingestion	Proton-pump inhibitor	NA	1	Yes	NA	NA
**Nakatani *et al.*[77] (2017)**	Case report	1	70 / M	Granulomatosis with polyangiitis (Wegener granulomatosis); mizoribine, steroids	No	2 ATA, 90 min, daily	13	Yes	Yes	36
**Spyres *et al.*[78] (2017)**	Case report	1	45 / M	Hydrogen peroxide ingestion	NA	3.0 ATA, NA	1	Yes	Yes	NA
**Aslam *et al.*[26] (2018)**	Case report	1	32 / M	Recent emergency open appendectomy	Elemental diet, antibiotics	NA	NA	No	No	Surgery
**Tamura *et al.*[27] (2018)**	Case report	1	73 / M	Appendectomy (50 yrs before), recent post-surgery ileus	Normobaric oxygen, bowel rest, panthetine, prostaglandin F2 alpha, long-acting octreotide	NA	9	No	No	Surgery
**Tsuboi *et al.*[79] (2018)**	Case report	1	14 / F	Hydrogen peroxide ingestion	NA	2.0 ATA, 120 min	1	Yes	Yes	NA
**Youssef *et al.*[80] (2018)**	Case report	1	52 / M	Hydrogen peroxide ingestion, gastro-oesophageal reflux disease	No	NA	NA	Yes	NA	NA
**Ghabach *et al.*[81] (2020)**	Case report	1	22 / M	Hydrogen peroxide ingestion	NA	NA	NA	Yes	Yes	NA
**Toyota *et al.*[18] (2020)**	Case report	1	59 / M	Decompression sickness with mesenteric venous thrombosis and pneumatosis in the intestines	No	USN TT6**	1	No	No	Surgery
**Yu *et al.*[19] (2020)**	Case report	1	42 / M	Decompression sickness with mesenteric venous thrombosis and pneumatosis in the liver, mesentery and intestines	NA	USN TT6 / USN TT6A (2 sessions) ** -> 2.0-2.2 ATA (6 sessions)	8	Yes	Yes	3

ATA, atmosphere absolute; CR, complete response; F, female; FU, follow-up; HBOT, hyperbaric oxygen therapy; NA, not available; O_2, oxygen; M, male; Mos, months; N, no; NSAIDs, nonsteroidal anti-inflammatory drugs; PBSCT, peripheral blood stem cell transplantation; PCI, pneumatosis cystoides intestinalis; Pts., patients; US, United States; wk., week; Y, yes; Yrs, years. * Comex 30 (heliox 50/50) Table: the protocol starts with recompression to 3 ATA using helium-oxygen (50/50) as a treatment gas mixture. The decompression that follows includes stops at 2.4, 1.8 and 1.2 ATAs. From 1.8 ATA to the surface the treatment gas is 100% oxygen. During the decompression schedule several 5-min air intervals have been included to reduce the risk of oxygen toxicity. ** US Navy Treatment Table 6 (USN TT6): the protocol starts with rapid compression to 2.8 ATA on air, followed by three 20-min periods on oxygen each followed by 5-min air intervals. Then, ascent to 1.9 ATA on oxygen is performed in 30 min, after which two blocks of 15-min air and 60-min oxygen follow. The ascent to the surface is performed on oxygen in 30 min. The total treatment takes 4 hr 48 min.
